# In Silico and In Vitro Evaluation of Bioactive Constituents Isolated from *Ziziphus oxyphylla* for the Treatment of Diabetes

**DOI:** 10.3390/biom16050700

**Published:** 2026-05-09

**Authors:** Muhammad Zia-Ul-Haq, Waqar Ahmad Kaleem, Syeda Ayesha Farhana, Syed Uzair Ali Shah, Abdul Rehman, Saqib Khan, Iffat Naz

**Affiliations:** 1 Department of Pharmacy, University of Swabi, Ambar, Swabi 94640, Khyber Pakhtunkhwa, Pakistan; mzia382@gmail.com (M.Z.-U.-H.); uzair@uoswabi.edu.pk (S.U.A.S.); dr.saqib0099422@gmail.com (S.K.); 2 Department of Pharmaceutics, College of Pharmacy, Qassim University, Buraydah 51452, Saudi Arabia; a.farhana@qu.edu.sa; 3 Department of Microbiology, Kohat University of Science and Technology (KUST), Kohat 26000, Khyber Pakhtunkhwa, Pakistan; abdulrehman@kust.edu.pk; 4 Department of Biology, College of Science, Qassim University, Buraydah 51452, Saudi Arabia

**Keywords:** cyclopeptide alkaloids, *Z. oxyphylla*, antidiabetic activity, molecular docking, drug discovery, α-amylase inhibition, dipeptidyl peptidase-4 inhibition

## Abstract

*Zizyphus oxyphylla* has been traditionally used in the management of metabolic disorders, and cyclopeptide alkaloids isolated from its roots have been structurally characterized, although their antidiabetic potential remains insufficiently explored. This study evaluated the antidiabetic activity of three previously isolated cyclopeptide alkaloids, Oxyphylline-D (**1**), Nummularine-C (**2**), and Nummularine-R (**3**), using integrated in silico molecular docking and in vitro enzyme inhibition assays. Molecular docking was performed against key diabetes-related enzymes, including α-amylase, α-glucosidase, and dipeptidyl peptidase-4, followed by validation through enzyme inhibition assays. All compounds demonstrated favorable binding affinities toward the selected targets, with compound **2** showing the most consistent performance across enzymes. In vitro results support these findings, where compound **2** exhibited inhibitory activity with IC_50_ values of 102.97 µg/mL (187.71 µM) against α-amylase and 28.87 µg/mL (52.62 µM) against dipeptidyl peptidase-4, comparable to standard inhibitors. Overall, the results indicate that cyclopeptide alkaloids from *Z. oxyphylla* possess significant multi-target antidiabetic potential, with Nummularine-C emerging as a promising candidate for further pharmacological development. These findings provide experimental support for computational predictions and reinforce the relevance of combining in silico and in vitro approaches for identifying bioactive natural products with potential therapeutic value in diabetes management, drug discovery pipelines, and development.

## 1. Introduction

Diabetes mellitus, commonly known as diabetes, is a chronic metabolic disorder characterized by persistently elevated blood glucose levels due to either insufficient insulin production or ineffective insulin utilization [1,2]. The global burden of diabetes continues to escalate, with 589 million adults affected in 2024 (44% undiagnosed) and projections reaching 853 million by 2050 [3]. Concurrently, 1.1 billion individuals exhibit impaired glucose regulation [3], amplifying type 2 diabetes risk. The economic impact exceeded USD 1.015 trillion in 2024 [3], underscoring the need for novel therapeutic strategies.

Current pharmacological treatments for diabetes include insulin and various classes of oral hypoglycemic agents such as α-glucosidase inhibitors [4], biguanides, sulfonylureas, meglitinide analogs, dipeptidyl Peptidase-4 (DPP-4) inhibitors, thiazolidinediones, GLP-1 receptor agonists, and SGLT-2 inhibitors [5]. However, the clinical use of these agents is frequently limited by adverse effects. For example, metformin, a first-line biguanide, commonly causes gastrointestinal disturbances and is contraindicated in patients with advanced renal impairment due to the risk of lactic acidosis. Caution is also warranted in individuals with severe hepatic or cardiac dysfunction [6]. Thiazolidinediones, which enhance insulin sensitivity, may induce weight gain, fluid retention, and heart failure, and are contraindicated in similar comorbid conditions [7,8]. Rosiglitazone has been associated with an increased risk of myocardial infarction, while pioglitazone is restricted in certain countries due to its potential link to bladder cancer [9,10]. In contrast, herbal medicines, endorsed by the World Health Organization (WHO), are increasingly considered safer alternatives to synthetic drugs due to their lower side effect profiles [11,12]. Several marketed polyherbal formulations, such as Diabetics and Diabet, include a variety of plant-derived constituents with antidiabetic properties and are often recommended alongside lifestyle modifications [5].

To date, over 13,000 secondary metabolites have been identified from medicinal plants, and they are classified into chemical groups such as glycosides, flavonoids, phenolics, terpenoids, xanthones, tannins, saponins, and alkaloids [13,14]. Many of these phytochemicals have been shown to exert antidiabetic effects [15]. Alkaloids are particularly notable due to their structural diversity and multi-target therapeutic potential. For instance, berberine (an isoquinoline alkaloid) activates AMP-activated protein kinase (AMPK) pathways, while indole alkaloids have been reported to enhance insulin secretion [13]. Recent studies have further emphasized the therapeutic potential of plant-derived bioactive compounds in diabetes management, particularly due to their multi-target mechanisms and improved safety profiles [16,17,18].

Plant-derived alkaloids, including indole, isoquinoline, terpenoids, and amino alkaloids, possess anti-diabetic properties. Examples include indole alkaloids from *Catharanthus roseus*, isoquinoline alkaloids like palmatine and berberine, non-terpenoid alkaloids from *Nigella glandulifera* seeds, and Aegeline from *Aegle marmelos* bark [19,20]. However, the structure–activity relationships (SAR) of cyclopeptide alkaloids, particularly regarding their effects on insulin signaling pathways and glucose transporter regulation, remain largely underexplored [21]. Based on these considerations, the present study was conducted to evaluate the antidiabetic potential of the crude root extract of *Ziziphus oxyphylla* and selected isolated cyclopeptide alkaloids, with the aim of providing a scientific rationale for the ethnopharmacological use of this plant in diabetes management.

## 2. Materials and Methods

### 2.1. Plant Material, Extraction, Fractionation, and Isolation

The extraction, fractionation, isolation, and structural elucidation of secondary metabolites from *Z. oxyphylla* Edgew roots were previously accomplished and reported by our group, Kaleem et al. [22]. These studies employed methanolic extraction, solvent partitioning, and silica gel chromatographic techniques, leading to the isolation of cyclopeptide alkaloids. The structures of the isolated compounds Oxyphylline-D (**1**), Nummularine-C (**2**), and Nummularine-R (**3**) were established using comprehensive spectroscopic analyses (^1^H NMR, ^13^C NMR, HR-ESI-MS) [23].

The current study does not involve re-extraction or re-isolation; rather, it is focused on the biological evaluation of these previously characterized compounds through in silico and in vitro assays.

### 2.2. In Silico Screening

#### 2.2.1. Protein and Ligand Preparation

Three-dimensional X-ray crystal structures of α-amylase (PDB ID: 3BAJ [24]), α-glucosidase (PDB ID: 1BGA [25]), and dipeptidyl peptidase-4 (DPP-4; PDB ID: 4A5S [26]) were retrieved from the RCSB Protein Data Bank (https://www.rcsb.org/). Protein structures were prepared using AutoDock Vina Tools v1.5.7 [27], where all crystallographic water molecules and heteroatoms were removed, polar hydrogens were added, and Gasteiger charges were assigned [28]. The test ligands (Compound **1**, Compound **2**, and Compound **3**) were drawn using ChemDraw version 2021, converted into 3D conformations using Avogadro [29], and energy-minimized using the MMFF94 force field [30]. The standard inhibitors Acarbose (PubChem CID: 9811704) and Diprotin-A (PubChem CID: 94701) were downloaded from the PubChem [31] and processed identically.

#### 2.2.2. Molecular Docking Protocol

Molecular docking was performed using AutoDock Vina [32,33]. Grid boxes were defined around the catalytic sites based on co-crystallized ligand coordinates. Flexible ligand docking was enabled, and redocking of co-crystallized ligands was carried out for protocol validation. RMSD values < 2.0 Å were considered acceptable [34]. Docking scores (kcal/mol) were used to select the best binding poses. Standard inhibitors (Acarbose for α-amylase and α-glucosidase, Diprotin-A for DPP-4) were included as positive controls. However, no known inactive compounds were included as negative controls in this study.

#### 2.2.3. Visualization and Interaction Analysis

Docked complexes were visualized using PyMOL v2.5 (Schrödinger, LLC, New York, NY, USA) [35] and LigPlot+ [36] to evaluate hydrogen bonding, hydrophobic contacts, and aromatic stacking interactions [37]. The number and type of interactions between ligands and key catalytic residues were documented for comparative analysis. Interacting residues were arranged by residue number to standardize reporting. Special attention was directed toward interactions with experimentally validated catalytic or substrate-binding residues to assess potential inhibitory mechanisms [28,34].

### 2.3. In Vitro Enzymatic Assays

The in vitro inhibitory activities of the tested samples (crude extract, fractions, and isolated compounds) were evaluated against targeted enzymes (α-amylase, α-glucosidase, and DPP-4).

#### 2.3.1. α-Amylase Inhibitory Assay

The inhibitory activity of the tested samples (crude extract, fractions, and isolated compounds) against α-amylase was assessed using the method described by Sagbo and van de Venter [38], with slight modifications. In brief, 5 μL of pancreatic enzymes was mixed with 30 μL of the sample at different concentrations (31.25–1000 μg/mL) in 96-well microplates. After that, the solution was incubated at 37 °C for 10 min; during this period, 20 μL of starch solution was added to start the reaction and was then kept at that temperature for an additional 30 min. To finish this reaction, 10 μL of 1000 mM hydrochloric acid was mixed with iodine reagent (75 μL). A blank was prepared using phosphate buffer (pH 6.8) in place of the tested sample, and acarbose was used as a positive control. The absorbance was then measured at 580 nm using a UV-visible spectrophotometer, and the formula below was used to calculate the percentage inhibition of α-amylase enzymes.

$$\%\;inhibition=(1-\frac{Absorbance\;of\;sample}{Absorbance\;of\;control})\times 100$$

#### 2.3.2. α-Glucosidase Inhibitory Assay

The inhibitory activity of the tested samples (crude extract, fractions, and isolated compounds) against α-glucosidase was evaluated using a previously described method by Ahamad [34] with slight modifications. The enzyme solution was prepared by mixing 120 µL of potassium phosphate buffer (pH 6.8) with 20 µL of α-glucosidase at 0.50 U/mL. A 1 mol/L solution of p-nitrophenyl-α-D-glucopyranoside (PNPG) was prepared in the same buffer as the substrate. Test samples were prepared at concentrations ranging from 31.25 to 1000 µg/mL. Then, 10 µL of each test sample was mixed with 20 µL of enzyme solution and incubated at 37 °C for 10 min. Subsequently, 20 µL of the substrate solution was added, and the mixture was incubated again at 37 °C for another 10 min. The reaction was terminated by adding 80 µL of 100 mmol/L sodium carbonate solution. Absorbance was measured at 405 nm using a UV–visible spectrophotometer. Blanks were prepared using deionized water instead of the test sample or enzyme, and acarbose was used as a positive control. The percentage inhibition of α-glucosidase activity was calculated using the following formula:

$$\%\;inhibition=(1-\frac{Absorbance\;of\;sample}{Absorbance\;of\;control})\times 100$$

#### 2.3.3. DPP-4 Inhibitory Assay

The DPP-4 inhibitory potential of the tested samples (crude extract, fractions, and isolated compounds) was evaluated following the method described by Al-masri [39], with minor modifications. All assays were conducted in triplicate using a 96-well microplate under controlled conditions (37 °C; pH 6.8–7.5) to simulate physiological environments.

To prepare working solutions, 10 mg of each tested sample was dissolved in 20 mL of demineralized water to yield a stock concentration of 25 × 10^−3^ g/mL (500 µg/mL). From this stock, working concentrations of 0.625, 2.5, 10, and 20 × 10^−3^ g/mL (equivalent to 12.5, 50, 200, and 400 µg/mL, respectively) were prepared. A 20 µL aliquot of each was diluted with Tris-HCl buffer (0.05 mol/L, pH 7.5) to a final volume of 35 µL per well, resulting in test concentrations of 0.5, 2, 8, and 16 × 10^−3^ g/mL (i.e., 2.5, 10, 40, and 80 µg/mL, respectively) in a total reaction volume of 100 µL.

Fifteen microliters of DPP-4 enzyme (50 µg/µL or 50 × 10^3^ g/mL) were added to initiate the reaction by cleaving the chromogenic substrate, Gly-Pro-p-nitroanilide (GPPN, 0.2 mol/m^3^ or 200 µM), which is hydrolyzed into p-nitroaniline (p-NA). The reaction mixture was pre-incubated for 5 min at 37 °C to facilitate enzyme–inhibitor binding. Then, 50 µL of GPPN solution was added, and the mixture was incubated for an additional 30 min at 37 °C. The reaction was terminated by adding 25 µL of 25% acetic acid. Absorbance was measured at 410 nm.

Diprotin A was used as the standard reference inhibitor. It was tested at concentrations of 0.4, 0.8, 1.6, 3.2, 6.4, and 12.8 × 10^−3^ g/mL (equivalent to 0.2, 0.4, 0.8, 1.6, 3.2, and 6.4 µg/mL), following the same protocol for comparative analysis. The percentage inhibition of DPP-4 activity was calculated using the following equation:

$$\%\;inhibition=(1-\frac{Absorbance\;of\;sample}{Absorbance\;of\;control})\times 100$$

### 2.4. Statistical Analysis

All enzyme inhibition assays were conducted in triplicate, and results are expressed as mean ± standard deviation. IC_50_ values were calculated using non-linear regression (dose–response curve) in GraphPad Prism version 8.0. Molecular docking was initially performed using AutoDock Vina Tools (version 1.5.7) [27], and results were visualized using Discovery Studio Visualizer [40].

## 3. Results

### 3.1. Structure Elucidation of the Isolated Compounds

The phytochemical investigation of *Z. oxyphylla* roots has been previously reported by our group, Kaleem [23]; Nisar et al. [41], resulting in the isolation and structural characterization of several cyclopeptide alkaloids.

**Figure 1:**
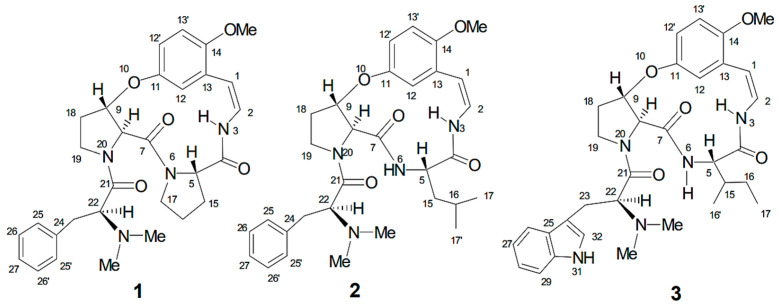
Chemical structures of isolated compounds **1**–**3** from *Z. oxyphylla* roots reported previously.

In the present study, three previously identified compounds, Oxyphylline-D (**1**), Nummularine-C (**2**), and Nummularine-R (**3**), were selected for further biological evaluation (Figure 1). The structures and purity of these compounds were consistent with previously reported data, and therefore no re-characterization was undertaken.

### 3.2. Computational Docking Studies

#### 3.2.1. Validation of Docking Protocol with Control Compounds

To evaluate the reliability of the docking protocol, standard inhibitors were docked with their corresponding target enzymes. Acarbose was selected as the control for α-amylase and α-glucosidase, while Diprotin-A was used for DPP-4. Redocking of the native ligands yielded RMSD values below 2.0 Å, confirming the reproducibility and accuracy of the docking approach.

**Figure 2:**
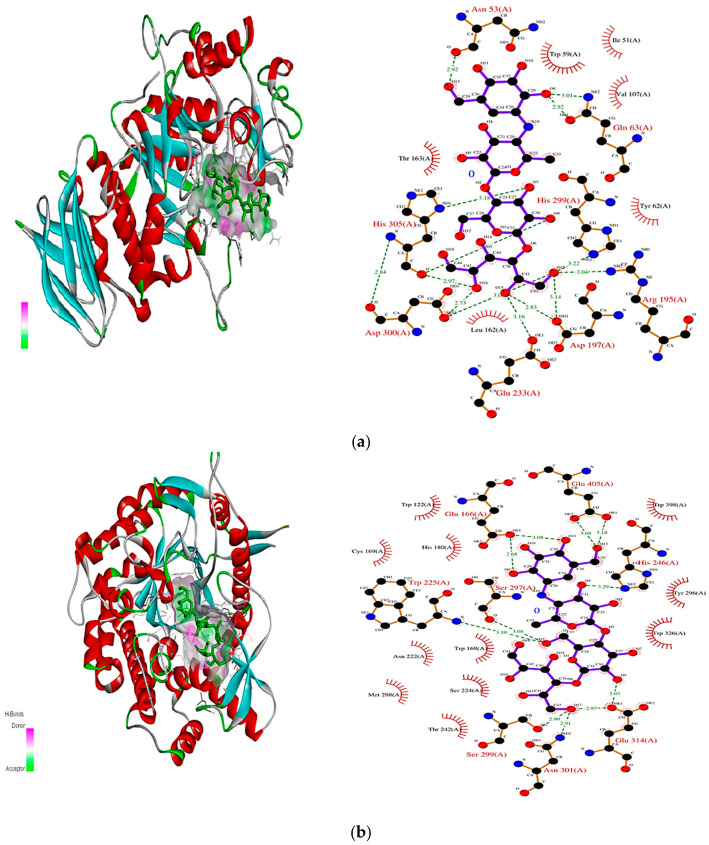

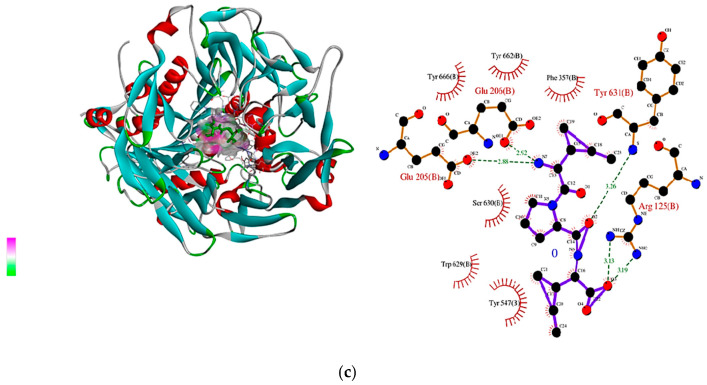
(**a**) Molecular docking analysis of acarbose with α-amylase (PDB ID: 3BAJ). Three-dimensional representation of acarbose within the active site of α-amylase, highlighting the binding pocket. Two-dimensional interaction diagrams show key hydrogen bonding and hydrophobic interactions with catalytic residues, including Asp197, Glu233, and Asp300. (**b**) Molecular docking analysis of acarbose with α-amylase (PDB ID: 1BGA). Three-dimensional representation showing acarbose bound within the active site of the enzyme. Two-dimensional interaction diagram illustrating key hydrogen bonding and hydrophobic interactions with surrounding amino acid residues, including Glu166, His246, Ser297, and Glu314, highlighting the stability of the ligand–protein complex. (**c**) Molecular docking analysis of Diprotin A with DPP-4 (PDB ID: 4A5S). Three-dimensional representation showing Diprotin A bound within the active site of DPP-4. Two-dimensional interaction diagram illustrating key hydrogen bonding and hydrophobic interactions with surrounding amino acid residues, including Glu205, Glu206, Tyr631, and Arg125, contributing to the stabilization of the ligand–protein complex.

(a) Acarbose with α-Amylase (PDB ID: 3BAJ): Acarbose exhibited a binding energy of −7.0 kcal/mol and formed 17 hydrogen bonds (H-bonds) within the enzyme’s active site. Key interactions were observed with Thr163, Lys200, Glu233, and His299, supported by van der Waals and hydrophobic interactions involving Trp59 and Tyr62 (Figure 2a).
(b) Acarbose with α-Glucosidase (PDB ID: 1BGA): The binding energy was −8.1 kcal/mol, with seven hydrogen bonds mainly involving Glu166, His121, and Trp406. Stabilizing interactions with Glu352 and Glu405 included van der Waals and aromatic contacts (Figure 2b).
(c) Diprotin-A with DPP-4 (PDB ID: 4A5S): Diprotin-A showed a binding energy of −7.2 kcal/mol, forming hydrogen bonds with Tyr62, Arg125, Ser630, and Glu205. Hydrophobic and π–alkyl interactions were noted with Phe357, Tyr666, and Trp629, along with one unfavorable donor–donor interaction (Figure 2c).

#### 3.2.2. Docking of Isolated Compounds

The binding affinities and interaction profiles of three cyclopeptide alkaloids, Oxyphylline-D (**1**), Nummularine-C (**2**), and Nummularine-R (**3**), were evaluated against three key enzymes, namely α-amylase (PDB ID: 3BAJ), α-glucosidase (PDB ID: 1BGA), and DPP-4 (PDB ID: 4A5S), and compared with standard inhibitors:

(a) Compounds **1**–**3** with α-Amylase (PDB ID: 3BAJ): Among the three isolated compounds, compound **3** exhibited the lowest (most negative) binding energy of −8.6 kcal/mol, indicating the strongest binding affinity. It was followed by compound **2** (−8.1 kcal/mol) and compound **1** (−7.1 kcal/mol). Compound **3** displayed favorable alignment within the enzyme’s active site, forming a hydrogen bond with Thr163 and hydrophobic interactions involving Ile51, Asn53, Trp59, Tyr62, Gln63, Val107, Ser108, Ser112, and Leu165. The binding affinity of compound **3** was superior to that of the standard acarbose (−7.0 kcal/mol), indicating its potential as an effective α-amylase inhibitor (Table 1).
(b) Compounds **1**–**3** with α-Glucosidase (PDB ID: 1BGA): In the case of α-glucosidase, compound **2** showed the highest binding affinity (−9.3 kcal/mol), outperforming both compound **3** (−8.1 kcal/mol) and compound **1** (−7.8 kcal/mol), as well as the standard acarbose (−8.1 kcal/mol). Compound **2** formed a key hydrogen bond with His180 and exhibited extensive non-covalent interactions, including π–π and van der Waals interactions with catalytically important residues such as Glu314, Ser297, His246, Trp168, Glu166, Trp406, Trp122, Glu405, Trp326, Ile324, Glu408, Leu173, Val79, Val223, and Ser224, suggesting strong stabilization within the binding pocket (Table 1).
(c) Compounds **1**–**3** with DPP-4 (PDB ID: 4A5S): For DPP-4 inhibition, compound **2** again demonstrated the most favorable binding energy (−10.1 kcal/mol), followed by compound **1** (−9.8 kcal/mol) and compound **3** (−9.0 kcal/mol). Compound **2** formed two critical hydrogen bonds with Tyr752 and Lys554, along with multiple stabilizing interactions involving residues such as Ala743, Tyr48, Ala564, Asn562, Trp563, Leu561, Trp627, Asp545, Val546, Ser630, Trp629, His748, and Gly741. Importantly, all tested compounds exhibited significantly stronger binding affinities compared with the standard Diprotin A (−7.2 kcal/mol) (Table 1).

**Table 1:** Molecular docking results of isolated compounds and standard against α-Amylase (PDB ID: 3BAJ), α-Glucosidase (PDB ID: 1BGA), and DPP-4 (PDB ID: 4A5S).

Compound	α-Amylase (3BAJ)			α-Glucosidase (1BGA)			DPP-4 (4A5S)		
	ΔG (kcal/mol)	Key H-Bond Residues	Other Residues Bonds	ΔG (kcal/mol)	Key H-Bond Residues	Other Residues Bonds	ΔG (kcal/mol)	Key H-Bond Residues	Other Residues Bonds
**1**	−7.1	Asn399, Arg10	Gly9, Val400, Ile396, Pro34, Ala33, Val89, Gln7, Gln8	−7.8	Lys342	Lys236, Ala226, Ile243, Ala240, Tyr339, Gln341, Trp225, Pro228	−9.8	Tyr631	Glu205, Tyr662, Arg125, Ser209, Phe357, Glu206, Arg669, Arg358, Ser630, Trp629, Val546, Lys554, Tyr547
**2**	−8.1	Ser289	His331, Glu282, Pro332, Asp290, Arg252, Pro4, Arg10, Thr11, Asp402, Phe335, Trp280, Tyr333	−9.3	His180	Glu314, Ser297, His246, Trp168, Glu166, Trp406, Trp122, Glu405, Trp326, Ile324, Glu408, Leu173, Val79, Val223, Ser224	−10.1	Tyr752, Lys554	Ala743, Tyr48, Ala564, Asn562, Trp563, Leu561, Trp627, Asp545, Val546, Ser630, Trp629, His748, Gly741
**3**	−8.6	Thr163	Ile51, Ser112, Ser108, Val107, Trp59, Tyr62, Leu165, Gln63, Asn53	−8.1	Arg384	Phe4, His383, Val380, Gln381, Arg331, Glu335	−9.0	Arg125, Ser630, His740, Asp708, Lys554	Tyr666, Tyr631, Tyr662, Val711, Tyr547, Gly741, Glu206, Glu205
Standard	−7.0 (Acarbose)	Asn53, Thr163, Gln63, His299, His305, Asp300, Asp197, Glu233, Arg195, Leu162	Trp59, Val107, Ile51, Tyr62, Leu162	−8.1 (Acarbose)	Ser297, Glu405, Glu166, His246, Glu314, Ser299, Asn301, Ser224	Trp122, Trp225, Trp168, Trp398, Tyr296, Trp326, Thr242, Met298, Asn222, His180, Cys169	−7.2 (Diprotin A)	Glu205, Glu206, Arg125, Tyr631	Trp629, Tyr547, Ser630, Tyr662, Tyr666, Phe357

### 3.3. In Vitro Anti-Diabetic Activity

#### 3.3.1. α-Amylase Activity

The α-amylase activity of the crude extract, n-hexane, chloroform (CHCl_3_), ethyl acetate, n-butanol, and aqueous fraction had IC_50_ values of 128.64, >1000, 120.41, 124.02, 337.66, and >1000 µg/mL, respectively. Chloroform displayed the greatest inhibitory potential among the tested fractions, with activity close to that of the standard drug acarbose (IC_50_ = 73.68 µg/mL).

**Figure 3:**
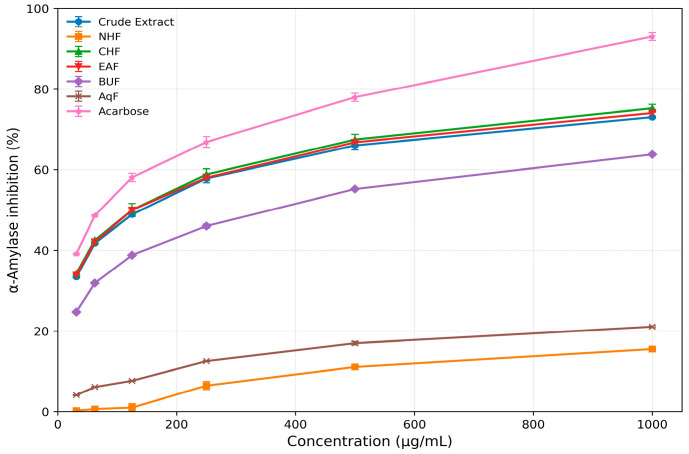
Dose–response curves illustrating the inhibitory activity of crude extract and solvent fractions (NHF, CHF, EAF, BUF, and AqF) of *Z. oxyphylla* roots against α-amylase, compared with standard acarbose. Data are presented as mean ± standard deviation (*n* = 3).

**Figure 4:**
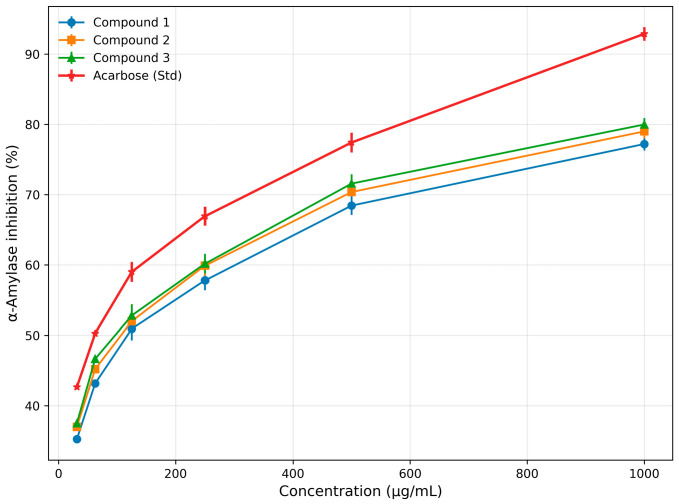
Dose–response curves illustrating the inhibitory activity of isolated compounds (Compound **1**, Compound **2**, and Compound **3**) of *Z. oxyphylla* roots against α-amylase, compared with standard acarbose. Data are presented as mean ± standard deviation (*n* = 3).

Similarly, the isolated cyclopeptide alkaloids such as compound **1**, compound **2**, and compound **3** had IC_50_ values of 119.01 (223.43 µM), 102.97 (187.67 µM), and 95.76 (162.93 µM) µg/mL, respectively. Compound **3** displayed the greatest inhibitory potential among the tested compounds, with activity close to that of the standard drug acarbose (IC_50_ = 63.05 µg/mL; 97.66 µM). The detailed inhibition data for all samples are provided in Table 2 and Table 3, with corresponding dose–response curves shown in Figure 3 and Figure 4.

**Table 2:** Percentage inhibition of α-amylase by the crude extract, subsequent fractions of *Ziziphus oxyphylla* root, and the standard drug acarbose.

S. No.	Conc. (µg/mL)	Crude Extract	NHF *	CHF *	EAF *	BUF *	AqF *	Std (Acarbose)
1	31.25	33.42 ± 0.22	0.25 ± 0.15	34.25 ± 0.25	33.87 ± 0.27	24.72 ± 0.22	4.12 ± 0.12	39.12 ± 0.22
2	62.5	41.76 ± 0.26	0.65 ± 0.15	42.50 ± 0.20	42.11 ± 0.21	31.90 ± 0.20	6.10 ± 0.10	48.65 ± 0.20
3	125	48.91 ± 0.34	1.00 ± 1.00	50.00 ± 1.61	49.98 ± 0.34	38.81 ± 0.10	7.66 ± 0.13	58.08 ± 1.02
4	250	57.81 ± 1.05	6.45 ± 1.02	58.80 ± 1.42	58.00 ± 0.12	46.01 ± 0.50	12.54 ± 0.14	66.85 ± 1.35
5	500	66.01 ± 1.06	11.09 ± 0.25	67.46 ± 1.31	66.79 ± 0.56	55.21 ± 0.20	16.98 ± 0.43	77.96 ± 1.01
6	1000	73.02 ± 0.27	15.53 ± 0.37	75.22 ± 0.97	74.05 ± 0.89	63.79 ± 0.30	21.05 ± 0.22	92.99 ± 0.97
IC_50_ (µg/mL)		128.64	>1000	120.41	124.02	337.66	>1000	73.68

Note: Data are presented as mean ± standard deviation, *n* = 3. IC_50_ values were calculated from dose–response curves using GraphPad Prism version 8.0. * NHF = n-hexane fraction; CHF = chloroform fraction; EAF = ethyl acetate fraction; BUF = n-butanol fraction; AqF = aqueous fraction.

**Table 3:** Percentage inhibition of α-amylase enzyme by isolated compounds of *Z. oxyphylla* and Std Acarbose.

S. No.	Conc. (µg/mL)	Compound 1	Compound 2	Compound 3	Std (Acarbose)
1	31.25	35.25 ± 0.05	36.98 ± 0.20	37.51 ± 0.25	42.66 ± 0.15
2	62.5	43.18 ± 0.12	45.22 ± 0.15	46.67 ± 0.30	50.28 ± 0.20
3	125	50.93 ± 1.63	52.01 ± 1.63	52.81 ± 1.62	59.01 ± 1.42
4	250	57.81 ± 1.40	59.91 ± 1.42	60.19 ± 1.40	66.95 ± 1.35
5	500	68.45 ± 1.32	70.38 ± 0.31	71.58 ± 1.32	77.43 ± 1.41
6	1000	77.21 ± 0.91	79.01 ± 0.99	79.99 ± 0.91	92.89 ± 0.97
IC_50_ (µg/mL)		119.01	102.97	95.76	63.05

Note: Data are presented as mean ± standard deviation, *n* = 3. IC_50_ values were calculated from dose–response curves using GraphPad Prism version 8.0.

#### 3.3.2. α-Glucosidase Inhibitory Activity

The α-glucosidase activity of the crude extract, n-hexane, chloroform (CHCl_3_), ethyl acetate, n-butanol, and aqueous fractions had IC_50_ values of 155.50, >1000, 141.39, 156.80, 373.69, and >1000 µg/mL, respectively. Chloroform displayed the greatest inhibitory potential among the tested fractions, with activity close to that of the standard drug acarbose (IC_50_ = 79.72 µg/mL).

Similarly, the isolated cyclopeptide alkaloid, compound **1**, had an IC_50_ value of 124.91 µg/mL (234.51 µM), showing notable inhibitory activity, though lower than the standard drug acarbose (IC_50_ = 75.31 µg/mL; 116.65 µM).

**Figure 5:**
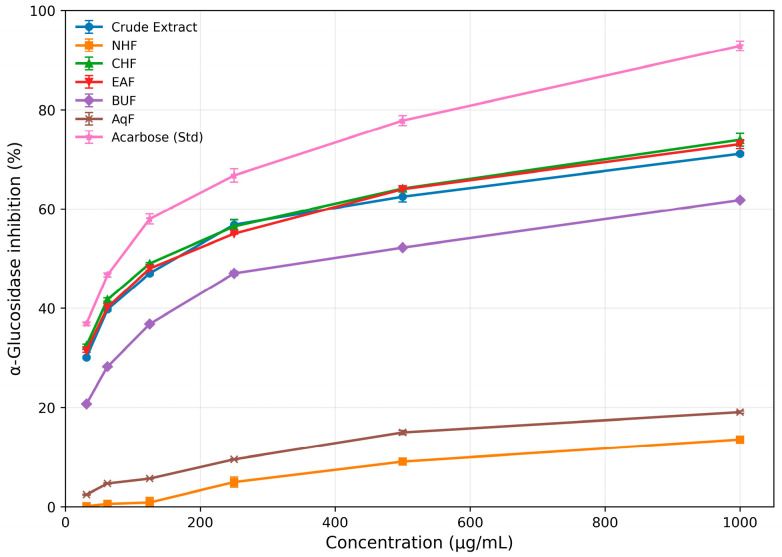
Dose–response curves illustrating the inhibitory activity of crude extract and solvent fractions (NHF, CHF, EAF, BUF, and AqF) of *Z. oxyphylla* roots against α-glucosidase, compared with standard acarbose. Data are presented as mean ± standard deviation (*n* = 3).

**Figure 6:**
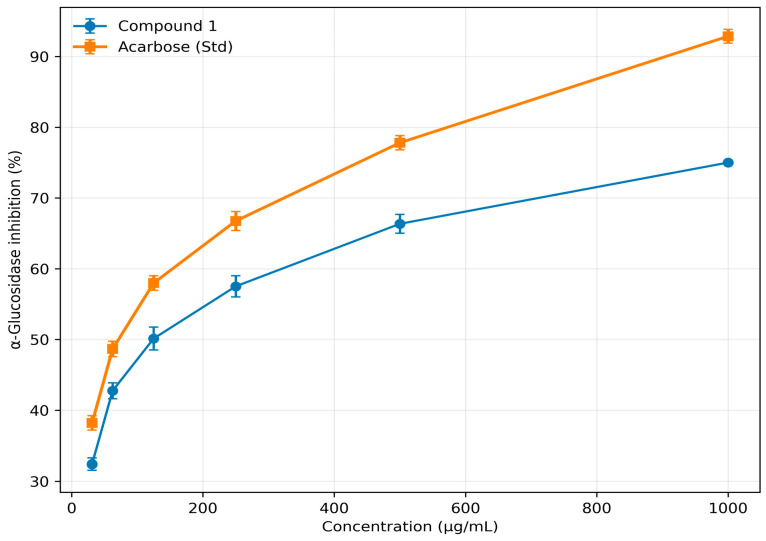
Dose–response curves illustrating the inhibitory activity of Compound **1** isolated from *Z. oxyphylla* roots against α-glucosidase, compared with standard acarbose. Data are presented as mean ± standard deviation (*n* = 3).

It should be noted that only compound **1** was experimentally evaluated for α-glucosidase inhibition in the present study, while data for compounds **2** and **3** were obtained from previously published literature [42]. Although these data provide useful comparative insights, differences in experimental conditions may limit direct comparability and should be interpreted with caution. The detailed inhibition data for all samples are provided in Table 4 and Table 5, with corresponding dose–response curves shown in Figure 5 and Figure 6.

**Table 4:** Percentage inhibition of α-Glucosidase by the crude extract, subsequent fractions of *Z. oxyphylla* root, and the Std drug (acarbose).

S. No.	Conc. (µg/mL)	Crude Extract	NHF *	CHF *	EAF *	BUF *	AQF *	Std. (Acarbose)
1	31.25	30.12 ± 0.24	0.12 ± 0.06	32.50 ± 0.26	31.44 ± 0.28	20.68 ± 0.21	2.45 ± 0.11	36.89 ± 0.34
2	62.5	39.76 ± 0.32	0.58 ± 0.15	41.73 ± 0.35	40.18 ± 0.29	28.25 ± 0.27	4.68 ± 0.13	46.70 ± 0.40
3	125	47.02 ± 0.34	0.87 ± 1.01	49.00 ± 0.18	47.98 ± 0.34	36.81 ± 0.12	5.66 ± 0.13	57.98 ± 1.02
4	250	56.81 ± 1.05	4.95 ± 1.02	56.46 ± 1.34	55.00 ± 0.12	47.01 ± 0.45	9.54 ± 0.14	66.75 ± 1.35
5	500	62.48 ± 1.06	9.09 ± 0.25	64.12 ± 0.25	63.99 ± 0.56	52.21 ± 0.21	14.98 ± 0.43	77.81 ± 1.01
6	1000	71.11 ± 0.27	13.53 ± 0.37	73.93 ± 1.27	73.05 ± 0.89	61.79 ± 0.32	19.05 ± 0.22	92.87 ± 0.97
IC_50_ (µg/mL)		155.5	>1000	141.39	156.8	373.69	>1000	79.72

Note: Data are presented as mean ± standard deviation, *n* = 3. IC_50_ values were calculated from dose–response curves using GraphPad Prism version 8.0. * NHF = n-hexane fraction; CHF = chloroform fraction; EAF = ethyl acetate fraction; BUF = n-butanol fraction; AqF = aqueous fraction.

**Table 5:** Percentage inhibition of α-Glucosidase enzyme by isolated compounds of *Z. oxyphylla* and Std Acarbose.

S. No.	Conc. (µg/mL)	Compound 1	Std (Acarbose)
1	31.25	32.40 ± 0.85	38.20 ± 1.02
2	62.5	42.75 ± 1.12	48.65 ± 1.09
3	125	50.13 ± 1.62	57.98 ± 1.02
4	250	57.51 ± 1.50	66.75 ± 1.35
5	500	66.35 ± 1.32	77.81 ± 1.01
6	1000	75.01 ± 0.21	92.87 ± 0.97
IC_50_ value		124.91	75.31

Note: Data are presented as mean ± standard deviation, *n* = 3. IC_50_ values were calculated from dose–response curves using GraphPad Prism version 8.0.

#### 3.3.3. DPP-4 Inhibitory Activity

The DPP-4 inhibitory activity of the crude extract, n-hexane, chloroform (CHCl_3_), ethyl acetate, n-butanol, and aqueous fractions had IC_50_ values of 41.76, >1000, 35.36, 38.36, 45.43, and 133.02 µg/mL, respectively. Chloroform displayed the greatest inhibitory potential among the tested fractions, with activity approaching that of the standard drug Diprotin A.

**Figure 7:**
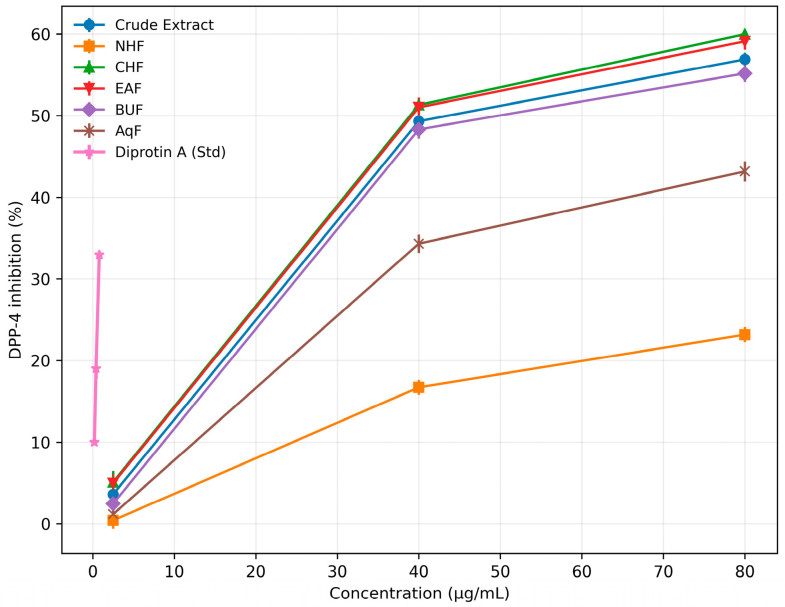
Dose–response curves illustrating the inhibitory activity of crude extract and solvent fractions (NHF, CHF, EAF, BUF, and AqF) of *Z. oxyphylla* roots against DPP-4, compared with standard Diprotin A. Data are presented as mean ± standard deviation (*n* = 3).

**Figure 8:**
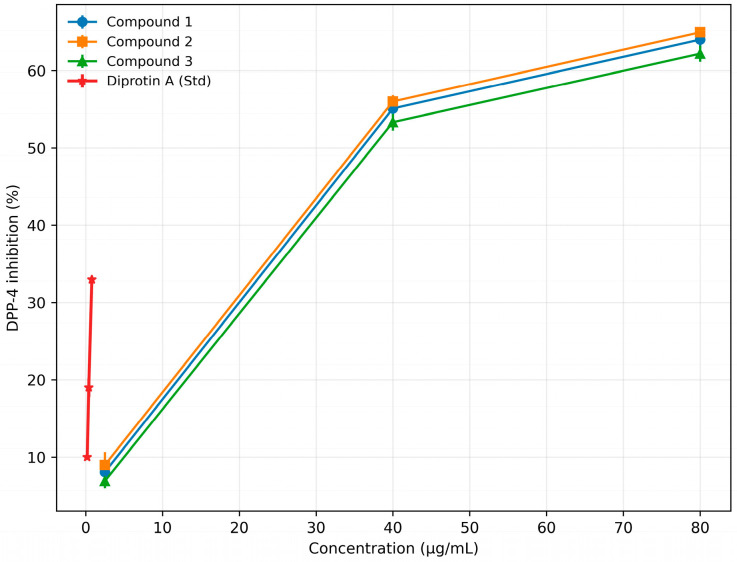
Dose–response curves illustrating the concentration-dependent inhibition of DPP-4 by isolated cyclopeptide alkaloids (Compound **1**, Compound **2**, and Compound **3**) from *Z. oxyphylla* roots, in comparison with the reference inhibitor Diprotin A. Data are presented as mean ± standard deviation (*n* = 3).

Similarly, the isolated cyclopeptide alkaloids, compound **1**, compound **2**, and compound **3**, had IC_50_ values of 30.51 (57.28 µM), 28.87 (52.62 µM), and 33.70 (57.34 µM) µg/mL, respectively. Among these, compound **2** exhibited the greatest inhibitory potential, with activity approaching that of the standard drug Diprotin A (IC_50_ = 1.46 µg/mL; 3.68 µM). The detailed inhibition data for all samples are provided in Table 6 and Table 7, with corresponding dose–response curves shown in Figure 7 and Figure 8.

## 4. Discussion

The phytochemical investigation of *Z. oxyphylla* has previously led to the isolation and structural characterization of several cyclopeptide alkaloids, including Oxyphylline-D (**1**), Nummularine-C (**2**), and Nummularine-R (**3**), as reported by our group [23,41,43,44]. Building upon these established findings, the present study was designed to explore the biological potential of these compounds through integrated in silico and in vitro approaches.

Molecular docking (Table 1) was performed prior to in vitro evaluation to predict binding affinities toward α-amylase, α-glucosidase, and DPP-4, using established protocols for enzyme–ligand interaction assessment [45,46]. Redocking experiments confirmed RMSD values below 2.0 Å, validating the docking methodology and confirming high accuracy and reproducibility [45,46]. Standard inhibitors such as acarbose for α-amylase and α-glucosidase, and Diprotin A for DPP-4 were used as positive controls to benchmark ligand binding predictions. All three alkaloids bound effectively within the enzyme active sites, with ΔG values generally more negative than those of the standards, indicating stronger predicted binding affinities. For α-amylase (PDB: 3BAJ), Nummularine-R exhibited the most negative binding energy (−8.6 kcal/mol), followed by Nummularine-C (−8.1 kcal/mol), both surpassing acarbose (−7.0 kcal/mol). For α-glucosidase (PDB: 1BGA), Nummularine-C showed the most negative ΔG (−9.3 kcal/mol), while for DPP-4 (PDB: 4A5S), Nummularine-C again ranked highest (−10.1 kcal/mol), exceeding Oxyphylline-D (−9.8 kcal/mol), Nummularine-R (−9.0 kcal/mol), and Diprotin A (−7.2 kcal/mol). These in silico results align with previous findings that structural dynamics influence stability and binding affinity of disease-associated targets [28]. Hydrophobic and π–π interactions with aromatic residues (Trp59, Trp406, Trp629) predominated over hydrogen bonding in complex stabilization. Although the tested compounds exhibited more favorable docking scores compared with standard inhibitors, their in vitro IC_50_ values indicate lower inhibitory potency. This discrepancy reflects a known limitation of molecular docking, which estimates binding affinity based on static structural models and does not fully account for protein flexibility, solvation effects, or kinetic parameters influencing biological activity. Thus, docking results should be considered as qualitative indicators of potential interactions rather than quantitative predictors of experimental potency.

In vitro assays confirmed many of the docking predictions (Table 2, Table 3, Table 4, Table 5, Table 6 and Table 7). For α-amylase, Nummularine-C (IC_50_ = 102.97 µg/mL; 187.67 µM) displayed the second-highest activity after Nummularine-R (95.76 µg/mL; 162.93 µM), approaching acarbose (63.05 µg/mL; 97.66 µM). For α-glucosidase, only Oxyphylline-D was tested in this study (124.91 µg/mL; 234.51 µM) because Nummularine-C and Nummularine-R values were previously reported (215.1 µM and 212.1 µM, respectively). Although assay conditions may differ, these indicate all three compounds inhibit α-glucosidase, with Nummularine-R strongest and Nummularine-C second strongest. For DPP-4, Nummularine-C was most active (28.87 µg/mL; 52.62 µM), closely approaching Diprotin A (1.46 µg/mL; 3.68 µM). Oxyphylline-D and Nummularine-R also showed substantial inhibition (30.51 µg/mL; 57.28 µM and 33.70 µg/mL; 57.34 µM, respectively). Across all enzymes, Nummularine-C ranked highest overall, showing no weak activity in any assay. It should be noted that only compound **1** was experimentally evaluated for α-glucosidase inhibition in the present study, while data for compounds **2** and **3** were obtained from previously published literature. Although these data provide useful comparative insights, differences in experimental conditions may limit direct comparability and should be interpreted with caution.

The structure–activity relationship (SAR) suggests that the conserved 14-membered macrocyclic ring forms the scaffold for binding, while variations in side chains modulate potency. Nummularine-C’s hydrophobic benzyl group and compact conformation likely enhance van der Waals and π–π stacking, favoring α-amylase and DPP-4 inhibition. Nummularine-R’s bulky dimethyltryptophan moiety may contribute to its strong α-glucosidase activity via extended pocket accommodation. Oxyphylline-D, with its methoxy-substituted aromatic ring, may have reduced hydrogen bonding potential, explaining its slightly weaker performance. These findings agree with earlier molecular docking studies that demonstrated how hydrophobic and π–π interactions with aromatic residues can significantly contribute to complex stabilization [47].

The alignment of docking and in vitro results underscores the potential of these cyclopeptide alkaloids as multi-target antidiabetic agents. Inhibiting α-amylase and α-glucosidase delays carbohydrate breakdown and glucose absorption [48], while DPP-4 inhibition prolongs the action of incretins (GLP-1 and GIP), stimulating insulin secretion and suppressing glucagon release [49]. This dual mechanism aids in postprandial glucose control and overall glycemic regulation in T2DM [50]. However, the present study did not include enzyme kinetic analyses (e.g., Lineweaver–Burk plots) or competition assays with standard inhibitors to determine the precise mode of inhibition (competitive, non-competitive, or mixed). Therefore, while molecular docking suggests potential interactions within catalytic sites, these findings should be interpreted as predictive rather than confirmatory. Future studies incorporating enzyme kinetics and binding assays will be essential to validate the proposed inhibition mechanisms and to confirm target engagement. Additionally, mechanistic interpretations based solely on docking analysis should be approached with caution, as complementary validation techniques such as molecular dynamics simulations, mutagenesis studies, or enzyme kinetics were not performed in this study.

Moreover, the present study did not investigate the effects of these alkaloids on specific intracellular diabetic signaling pathways, such as insulin secretion, GLUT4 translocation, or AMPK activation. The observed enzyme inhibition and docking interactions suggest possible modulation of these mechanisms, but further mechanisms and in vivo studies are warranted to confirm this.

While the findings are promising, both in silico and in vitro approaches have inherent limitations. Docking simulations may not fully account for protein flexibility or solvation effects, and in vitro systems lack the complexity of whole-organism physiology. Additionally, further enzyme kinetic studies, in silico ADMET profiling, in vitro toxicity, and in vivo pharmacokinetic and pharmacodynamic studies are essential to validate these preliminary results and to elucidate the full therapeutic potential of cyclopeptide alkaloids from *Z. oxyphylla* in the management of T2DM.

## 5. Conclusions

The present study extends previous phytochemical investigations of *Z. oxyphylla* by evaluating the biological potential of its previously isolated cyclopeptide alkaloids through integrated in silico and in vitro approaches. The findings demonstrate that Oxyphylline-D, Nummularine-C, and Nummularine-R possess significant inhibitory activity against key carbohydrate-metabolizing enzymes, including α-amylase, α-glucosidase, and DPP-4. Among the tested compounds, Nummularine-C consistently exhibited the most favorable binding affinities and strongest inhibitory potential across multiple targets, indicating its promise as a multi-target antidiabetic agent. The observed agreement between molecular docking and in vitro results further supports the reliability of the proposed mechanism of enzyme inhibition. Overall, this study provides scientific validation for the therapeutic relevance of *Z. oxyphylla* and highlights its cyclopeptide alkaloids as promising lead compounds for the development of novel antidiabetic agents. Future investigations should include enzyme kinetic studies, in silico ADMET profiling, and in vitro toxicity evaluation to better assess the pharmacokinetic behavior and safety profiles of these compounds.

## Figures and Tables

**Table 6:** Percentage inhibition of DPP-4 by the crude extract, fractions of *Z. oxyphylla* root, and Std Diprotin A.

S. No.	Conc. (µg/mL)	Crude Extract	NHF *	CHF *	EAF *	BUF *	AQF *	Conc. (µg/mL)	Std. (Diprotin A)
1	2.5	3.62 ± 1.03	0.41 ± 1.03	5.11 ± 1.37	4.96 ± 1.12	2.46 ± 0.93	1.16 ± 0.63	0.2	9.99 ± 0.12
2	40	49.32 ± 1.02	16.72 ± 0.91	51.32 ± 0.67	51.02 ± 1.22	48.32 ± 1.13	34.32 ± 1.13	0.4	19.00 ± 1.19
3	80	56.91 ± 0.83	23.18 ± 0.93	59.99 ± 0.39	59.12 ± 1.01	55.18 ± 1.03	43.18 ± 1.23	0.8	32.96 ± 0.23
IC_50_ (µg/mL)		41.76	>1000	35.36	38.36	45.43	133.02		1.46

Note: Data are presented as mean ± standard deviation, *n* = 3. IC_50_ values were calculated from dose–response curves using GraphPad Prism version 8.0. * NHF = n-hexane fraction; CHF = chloroform fraction; EAF = ethyl acetate fraction; BUF = n-butanol fraction; AqF = aqueous fraction.

**Table 7:** Percentage inhibition of DPP-4 enzyme by isolated compounds of *Z. oxyphylla* and Std Diprotin A.

S. No.	Conc. (µg/mL)	Compound 1	Compound 2	Compound 3	Conc. (µg/mL)	Std (Diprotin A)
1	2.5	8.07 ± 1.43	8.98 ± 1.67	6.91 ± 0.93	0.2	9.99 ± 0.12
2	40	55.09 ± 1.06	55.99 ± 0.83	53.32 ± 1.08	0.4	19.00 ± 1.19
3	80	64.01 ± 1.08	64.95 ± 0.60	62.19 ± 1.04	0.8	32.96 ± 0.23
IC_50_ (µg/mL)		30.51	28.87	33.70		1.46

Note: Data are presented as mean ± standard deviation, *n* = 3. IC_50_ values were calculated from dose–response curves using GraphPad Prism version 8.0.

## Data Availability

All data supporting the findings of this study are included within the article. Further details are available from the corresponding author upon reasonable request.
